# Evidence Supporting Selective Dorsal Rhizotomy for Treatment of Spastic Cerebral Palsy

**DOI:** 10.7759/cureus.3466

**Published:** 2018-10-19

**Authors:** TS Park, Matthew B Dobbs, Junsang Cho

**Affiliations:** 1 Neurological Surgery, Washington University School of Medicine, St. Louis Children's Hospital, St. Louis, USA; 2 Pediatric Orthopedic Surgery, Washington University School of Medicine, St. Louis Children's Hospital, St. Louis, USA

**Keywords:** cerebral palsy, gross motor function measure (gmfm), gross motor function classification system, physical therapy, selective dorsal rhizotomy

## Abstract

The objective of this review is to analyze the evidence supporting selective dorsal rhizotomy (SDR) for the treatment of spastic cerebral palsy (CP). We reviewed 85 outcome studies from 12 countries between 1990 and 2017. The published results are overwhelmingly supportive of SDR, and 39 studies form a basis for this review. Also included is some of the clinical experience of the senior author.

The results show that SDR plus postoperative physiotherapy (PT) improved gait, functional independence, and self-care in children with spastic diplegia. In adults with a follow-up of 20 to 28 years, the early improvements after childhood SDR were sustained and improved quality of life. Furthermore, majority of the adults who underwent SDR as children would recommend SDR to others. On the clinical side, while SDRs through multilevel laminectomies or laminoplasty were associated with spinal deformities (i.e., scoliosis, hyperlordosis, kyphosis, spondylolisthesis, spondylolysis, and nonhealing of laminoplasty), SDRs through a single level laminectomy prevented SDR-related spinal problems. The outcomes of SDR specific to spastic quadriplegia require further investigation because of the relatively small patient population with quadriplegia. Lastly, we found that SDR can prevent or reverse premature aging in adolescents and adults with spastic diplegia. In conclusion, the evidence supporting the efficacy of SDR is strong, and SDR is a well-established option for spasticity management in spastic CP.

## Introduction and background

In the last 30 years, selective dorsal rhizotomy (SDR) endured the test of time as a surgical treatment plan for spastic cerebral palsy (CP) patients as many countries have adopted the neurosurgical procedure. In 2010, a review panel at the National Institute for Health and Care Excellence in England concluded that the evidence on the efficacy of SDR is “adequate” [1]. In 2013, the National Health Service in England issued a policy statement, “There is moderate quality evidence that SDR plus physiotherapy in children resulted in significant improvement in spasticity and gross motor function over the 12-month follow-up” [2]. In 2017, the Health Quality Ontario of Canada reviewed studies of SDR between 1990 to 2016, and a review panel concluded, “Lumbrosacral dorsal rhizotomy and physical therapy effectively reduces lower-limb spasticity in children with spastic cerebral palsy and significantly improves their gross motor function and functional independence” [3]. There has been overwhelming evidence in favor of the SDR treatment for patients with CP and our own experiences also provide validation for the use of this procedure as a treatment plan.

## Review

Functional outcome in adults after childhood SDR

The natural history of spastic CP is a decline of mobility and function with aging. Many patients with spastic CP start to lose motor functions in adolescence and early adulthood partly due to spasticity. In our experience, the reduction of spasticity with SDR can prevent or even reverse premature aging (Videos 1, 2). If the post-SDR adult patients maintain or improve their pre-SDR walking and functional status, the outcomes will be beneficial for SDR patients. Since many patients who underwent SDR during childhood became adults in the last three decades, mobility and other functions of adults treated with childhood SDR deserve investigation. We studied 294 (36%) of 810 patients who underwent childhood SDR for spastic CP [4]. The follow-up period was 2-28 years, and the age at the last follow-up was 18 to 37 years. In this patient cohort, 84% had diplegia, 12% had quadriplegia, and 4% had triplegia. At the last follow-up, 87% of the 294 patients had Gross Motor Function Classification System (GMFCS) Levels I-III and 13% had GMFCS Levels IV and V. Concerning postoperative improvement, 30% of patients improved ambulatory function after SDR, and 53% had an ambulatory level similar to before surgery. Concerning patient perception of SDR, 83% would recommend SDR to other children with CP, 3% would not recommend the procedure, and 14% were unsure. A reason for being unsure was the inability to recall how impactful the SDR was for patients during childhood. We also examined living standards, education, employment, post-SDR treatment, pain, bladder function, and sensory changes. We concluded that the beneficial effects of childhood SDR extend to the quality of life in adulthood and ambulatory function without late side effects of surgery. Hurvitz et al. also surveyed 88 adolescents and adults who received SDR as children [5]. They reached similar conclusions regarding the improved quality of life as our study did.

**Video 1:** Effects of Selective Dorsal Rhizotomy (SDR) on Premature Aging Permission was given by the patients/parents to display patient identifying information in the videos.

**Video 2:** Effects of Selective Dorsal Rhizotomy (SDR) on Premature Aging Permission was given by the patients/parents to display patient identifying information in the videos.

Effects of SDR on functional outcome in 20-28 years

Our St. Louis group published ambulation outcomes for patients 20 to 28 years after SDR [6]. In a population of 316 patients who underwent SDR as children from 1987 to 1996, 95 (30%) patients between the ages of 23 to 37 participated in completing a survey. Spastic CP subtype consisted of diplegia in 79%, quadriplegia in 20%, and triplegia in 1%. At the last follow-up, 42% noted improved ambulation level; 42% had an ambulation level similar to the preoperative level; 14% stated decreased ambulation level. Before the procedure, 30% ambulated independently; 4% ambulated with crutches/canes; 44% ambulated with walkers, and 22% were nonambulatory. After the SDR procedure, 40% walked independently; 19% walked with crutches/canes; 44% walked with walkers, and 19% were non-ambulatory. Our study showed that 20 to 28 years after SDR, more patients walked independently or with crutches/canes and fewer patients walked with walkers. Regarding the perception of SDR among patients, 88% would recommend the surgery to others. If spasticity remained untreated, several patients would have noted deteriorated walking abilities in adulthood. The Cape Town group reported on locomotor function in 13 patients (age range 22-33 years) who underwent SDR as children 20 years earlier [7]. All patients had spastic diplegia and were ambulatory before surgery. Patients received gait analysis preoperatively, and one, three, 10, and 20 years after SDR. Knee and hip range of motion, cadence, and step length were assessed from the gait analysis. They concluded that 20 years after SDR the patients showed improved locomotor function compared to before SDR.

Effects of SDR on gross motor function in 5-17 years

The Montreal group reported Gross Motor Function Measure-88 (GMFM-88) scores at five, 10, and 15 years [8]. The study included 62, 57, and 14 patients at the three follow-up points. Due to the small number of children at the 15-year follow-up, it is difficult to assess the long-term motor functions. Nevertheless, mean GMFM-88 scores in each of the five motor subdomains significantly increased over baseline at each follow-up point. Of the ambulatory and nonambulatory groups, only the ambulatory group (GMFCS Levels I-III) made a significant gain in the gross motor function. Spasticity remained reduced at all follow-up points.

The Vancouver group reported a follow-up of 44 children undergoing SDR, which included both ambulatory and nonambulatory children with spastic CP [9]. The GMFM-66 scores were measured at five and 14 years of follow-up. In both groups, GMFM-66 scores increased at five-year follow-up, while the GMFM-66 scores were lower at the 14-year than the five-year follow-up. At the 14-year follow-up, mean scores in nonambulatory patients declined and were no longer distinct from baseline. In parallel with the change in motor function, quadriceps muscle strength increased over baseline in both groups (more in ambulatory patients than in nonambulatory patients at the five-year follow-up). Muscle strength remained significantly higher at the 14-year follow-up in ambulatory groups.

The Amsterdam group reported on their 10-year follow-up of 28 children who were all ambulatory before SDR [10]. Five years after SDR, mean GMFM-66 scores improved in 10 out of 28 children. Ten years after SDR, mean GMFM-66 scores improved in six out of 20 children. None of the children showed deterioration of gross motor function during the follow-up period. 

The Lund group reported five-year follow-up of 35 children with spastic diplegia [11]. Preoperative GMFCS Levels were l-lll in 19 children and IV-V in 16 children. They all received physiotherapy (PT) before/after surgery, and all children were evaluated preoperatively and at six, 12, 18, 36, 60 months postoperatively. In the ambulating group (GMFCS Levels l-lll), seven of the nine children gradually increased their motor goal scores over follow-up. In the assisted ambulation group (GMFCS lV-V), nine of the ten children increased their motor score. Overall, 84% (16/19) of the ambulating group improved their motor scores, and no major complications occurred postoperatively. It is important to note that during the five years of follow-up, 15 children (42%) had orthopedic surgery in the lower extremities. The authors concluded that “SDR is a safe and effective method for reducing spasticity permanently without major negative side effects. In combination with physiotherapy, in a group of carefully selected and systematically followed young children with spastic diplegia, it provides lasting functional benefits over a period of at least five years postoperatively.”

The Stockholm group reported functional outcomes 17 years after the SDR, and a total of 19 children with spastic diplegia underwent SDR in Stockholm between 1993 and 1997 [12]. Seventeen of them completed evaluations before SDR, and at three years, 10 years, and 15 to 19 years (mean 17 years) after surgery; however, the authors did not mention postoperative physical therapy protocol. They report that spasticity remained reduced over the baseline at all follow-up points. Mean GMFM-88 scores improved at three years but declined at 10 and 17 years. GMFM-88 scores before SDR and 17 years after SDR were largely comparable. Although the authors did not elaborate, a figure in the report indicates that the GMFM-88 remained improved in patients who had GMFCS l-ll and walked independently before surgery. During the 17 years after SDR, nearly all patients underwent a total of 68 orthopedic surgeries, including hip surgery in 10 patients. The authors concluded that SDR does not improve long-term motor functions nor prevents contractures.

Effects of SDR on gross motor function in two years or less

Eighteen studies evaluated patient follow-up of two years or less with GMFM. Three randomized trials compared motor functions of children with spastic diplegia who received SDR followed by PT against those who received only PT [13, 14, 15]. The study cohort was relatively small with 24, 29, and 38 children, respectively. All rhizotomies were performed through a multilevel lumbosacral laminectomy more than 20 years ago with varied techniques, and physical therapy protocols also varied across the three centers. The Vancouver and Toronto groups reported positive results [13, 15]. At 9 and 12 months after surgery, the SDR-PT group made more improvements in GMFM than the PT-only group, and all children in the two studies showed a reduction in lower limb spasticity. In contrast, the Seattle group found no significant improvement of GMFM at the one-year and two-year follow-up [15]. We attribute the lack of changes in the patients to cutting only 25% of the dorsal rootlets at surgery. Signs of spasticity persisted in 90% (19 of 21 children) of the study cohort after surgery. A subsequent meta-analysis of the three studies showed that the increased rate of dorsal rootlet cutting results in improvement of GMFM scores.

The St. Louis group reported a comparative non-randomized study that included a large study cohort [16]. The study examined 68 children with spastic diplegia and 40 non-disabled children. All diplegic children studied were independent or dependent ambulators (GMFCS levels l-lll). Thirty-one children were in the SDR-PT group, while 37 children were in the PT-only group. Forty nondisabled children were compared to the CP groups. SDR was performed through a single-level laminectomy at the L-1. After discharge, the SDR-PT group received PT from therapists in their hometowns, and the PT-only group received the same number of PT sessions. At eight and 20 months after surgery, GMFM scores, Gross Motor Activity Estimator (GMAE), spasticity, strength, and gait kinematics and speed were examined. The overall findings indicated that SDR offers a reduction of spasticity and gains in gross motor function, strength, and gait speed.

Effects of SDR on rate of orthopedic surgery

Our group reported three studies outlining the need for orthopedic surgery after SDR. In the first study, we examined the rate of orthopedic surgery pre- and post-SDR in a combined group of diplegic, quadriplegic, and hemiplegic children [17]. There were 116 diplegic, 58 quadriplegic, and four hemiplegic patients. The age at SDR ranged from two to nineteen years with follow-up intervals ranging from 24 to 70 months. They were divided into two age groups: two to four year olds (Group I, 54 children) and five to nineteen year olds (Group II, 124). The orthopedic surgeries included heel cord, iliopsoas, and hamstring release, femoral and ankle/ foot osteotomy, and other surgeries. At the time of the last follow-up review, 68 (38%) of 178 patients had undergone at least one orthopedic operation before and after SDR. The rate of operation was 22% in Group I and 45% in Group II. Those who underwent SDR during an earlier age had lower rates of subsequent orthopedic surgery than those who underwent the procedure at a later age.

In the second study, we examined orthopedic surgery after SDR in relation to ambulatory status and age at SDR in spastic diplegia for 158 diplegic child patients [18]. They were grouped into independent or assisted walkers, and the follow-up period was five to nine years. The age at time of SDR was as follows: two to three years old (Group 1), four to seven years (Group 2), and eight to fourteen years (Group 3). There were 59, 73, and 26 patients in Groups 1, 2, and 3, respectively. The overall rates of orthopedic surgery at last follow-up in all three age groups combined were 24% for independent walkers and 51% for assisted walkers. When divided by age group, the rates of orthopedic operations for independent and assisted walkers were 21 and 50%, 27 and 50%, and 21 and 58% for Groups 1, 2, and 3, respectively. Of the 158 patients studied, 127 underwent no orthopedic surgery before SDR. We found that the overall rate of orthopedic surgery five to nine years after SDR was 23% among independent walkers and 47% among assisted walkers. In all age groups combined, 25% of independent walkers and 44% of assisted walkers required orthopedic surgery in the follow-up period. Those who underwent SDR during ages two to three had a similar rate of orthopedic surgery, regardless of pre-SDR gait status (43% compared with 38%). By contrast, in the two groups of older patients (4-7 and 8-14 years), those who walked independently at the time of SDR underwent fewer orthopedic operations after rhizotomy than those who needed assistance walking.

In the third study, we examined the relationship between age and orthopedic surgery after SDR for spastic quadriplegia [19]. The study cohort consisted of 52 children who were followed for five to nine years post-SDR. Orthopedic procedures were recorded for two groups: patients two to five years of age (Group 1, 36 patients) and those six to fourteen years old (Group 2, 16 patients). Eleven percent of patients in the two- to five-year-old group and 38% in the six- to fourteen-year-old group had undergone orthopedic procedures before SDR. All orthopedic operations before SDR were soft-tissue procedures on the adductors, hamstrings, and heel cords. No patient in the two- to five-year-old age group underwent hamstring release before SDR. We examined the frequency of orthopedic operations among patients who had undergone no orthopedic surgery before SDR. The frequency was higher among the six- to fourteen-year-old age group than in the two- to five-year-olds (70% compared with 34%). Orthopedic operations after SDR, including derotational osteotomy for hip subluxation or femoral torsion, were more varied in the two- to five-year-old group. In the six- to fourteen-year-old group, the most common procedure after SDR was hamstring release. No patient in either group underwent spine surgery. In conclusion, early SDR (at the age of two to five years) may reduce the frequency of post-SDR orthopedic operations.

Effects of SDR on hip subluxation

Children with CP are at risk of developing progressive hip subluxation and dislocation. The risk of hip displacement is higher in nonambulatory spastic quadriplegic children. Hip instability after dorsal rhizotomy was first reported by Greene et al. in a small patient cohort consisting of six children, five of whom had spastic quadriplegia [20]. Park et al. reported the effects of SDR on hip subluxation in 67 diplegic children ranging from ages two to eleven with a follow-up period of 6-46 months [21]. At the last follow-up, hip migration remained unchanged in 75% of patients, improved in 17%, and worsened in 7%; thus, 93% of all hips were stable after SDR. We also studied 45 quadriplegic children aged two to nine years (follow up 7-50 months) [22]. Among the 90 hips examined, 9% improved, 80% remained unchanged, and 11% worsened, yielding a radiographic stability rate of 89%. The data indicates that SDR halts hip subluxation due to spastic diplegia or quadriplegia. Two other studies found a positive effect or no effect on hip joint subluxation rather than a deleterious effect [23, 24].

Effects of SDR on bladder function

Approximately a third of children with CP present with dysfunctional voiding symptoms. Three reports evaluated the impact of SDR on bladder function by comparing preoperative and postoperative symptoms, and urodynamic studies. Sweester et al. studied 34 children over the age of three, and video urodynamic study was performed in a subset of patients [25]. They found that nearly all patients with quadriplegia were incontinent before SDR, and none improved bladder control postoperatively. Almost half of the patients with diplegia who were incontinent before SDR gained continence postoperatively. They conclude that SDR can improve bladder capacity and control in diplegic children. Houle et al. reported on urodynamic studies in 40 children with a mean age of five [26]. Urodynamics were performed preoperatively in 22 patients, preoperatively and postoperatively in 13, and postoperatively in five. They concluded that at least half of the children with spastic CP had clinically silent bladder dysfunction. In the study by Chiu et al., 51 children had preoperative urodynamic studies, and 20 children had both preoperative and postoperative urodynamic studies [27]. They concluded that SDR significantly improved urgency, frequency, incontinence, and urodynamic bladder capacity in a significant proportion of children with spastic cerebral palsy.

Spinal deformities after SDR via multilevel laminectomy and laminoplasty

Until several years ago, SDR was performed most commonly through a multilevel lumbosacral laminectomy. Various reports documented the increased incidence of post-SDR spinal deformities after the multilevel laminectomy in children, i.e., back pain, scoliosis, kyphosis, lordosis, spondylolysis, spondylolisthesis, spinal stenosis, and non-healing laminoplasty. Peter et al. reported scoliosis in 16%, kyphosis in 5%, lordosis in 7%, and spondylolysis/spondylolisthesis in 9% of children, all of whom underwent five-level laminectomies [28]. Langerak et al. reported on 30 ambulatory patients who underwent L2-S1 laminectomies [29]. At 17 to 26 years after surgery, they compared preoperative and postoperative spine radiographs. At the last follow-up, scoliosis absent before rhizotomy occurred in 57% after rhizotomy, kyphosis absent before rhizotomy occurred in 7%, lordosis present in 21% before rhizotomy occurred in 40% after rhizotomy, and spondylolysis present in 18% before rhizotomy occurred in 37% after rhizotomy. Spondylolisthesis grade I occurred in one patient, spinal stenosis on MRI was present in 27%, and daily back pain occurred in 17%. Steinbok et al. studied 104 patients undergoing the various multilevel laminoplasties at T-12 to S-1, and one patient receiving the same level laminectomy [30]. At last follow up, 55% had scoliosis, and among them, five patients had improved scoliosis, while 26 patients exhibited worsened scoliosis. Additionally, four patients exhibited improved kyphosis, while 14 patients reported worsened kyphosis. Lastly, two patients showed improved lordosis, while 17 exhibited worsened lordosis. Golan et al. reported on 98 children who received multilevel L1-S1 laminoplasty, SDR and significant findings included the postoperative incidence of L5-S1 spondylolisthesis at 19% and spondylolysis of the L5 at 10% [31].

Spiegel et al. studied 77 ambulatory children treated by SDR with L1-L5 laminoplasty [32]. The mean radiographic follow-up was four years, and none of the children had either scoliosis or spondylolisthesis before rhizotomy. After rhizotomy, scoliosis was present in 17%, and spondylolisthesis was present in 12%. The degree of thoracic kyphosis and lumbar lordosis did not change after rhizotomy. In addition, healing of laminoplasty was examined. The percentage of patients with nonunion at either one or both sides of the laminoplasty was as follows: L1, 0%; L2, 1%; L3, 8%; L4, 35%; and L5, 46%. Twenty-nine percent had evidence of nonunion at both L4 and L5, and 6% at L3 through L5. Turi and Kalen reported on the wide L2 to S1 laminectomies on 46 children and one adult [33]. The follow-up ranged from two to nine years. They found 28 significant spinal deformities in 19 patients, scoliosis in 15 patients, lumbar hyperlordosis in seven patients, thoracic hyperkyphosis in five patients, and L4-5 spondylolisthesis in one patient. For the entire group, the risk of developing a structural spinal deformity was 36% after SDR.

St. Louis Children’s Hospital experience

From 1987 to 2018, we have performed SDR on 3,897 children and adults 2-49 years old (Tables 1-3). In 1991, we developed the SDR procedure via a single level laminectomy [34, 35]. The less invasive SDR procedure enabled a broad range of patients to benefit from receiving the surgery. Pre-existing spondylolisthesis, lordosis, mild scoliosis, and trunk weakness did not preclude our SDR procedure. We reported the beneficial short- and long-term surgical outcomes of our patients [4, 6, 16, 17-19, 21, 22, 36-39]. In 2009, we started a combined treatment with the less invasive SDR followed by less invasive orthopedic surgery to lengthen the hamstrings, gastrocnemius, or heel cord. The novel treatment rapidly improved walking and motor functions in our patients (Videos 2, 3). The outcome report is in preparation.

**Video 3:** Effects of Selective Dorsal Rhizotomy (SDR) on Motor Functions Permission was given by the patients/parents to display patient identifying information in the videos.

**Table 1:** Age at the Time of Selective Dorsal Rhizotomy (SDR) Surgery

Age (years)	Number of Patients
2-3	1,089 (28%)
4-6	1,660 (43%)
7-10	697 (18%)
11-16	311 (7%)
17-50	140 (4%)
Total	3,897 (100%)

**Table 2:** Ambulation at the Time of Selective Dorsal Rhizotomy (SDR) Surgery

Ambulation Level	Number of Patients
Independent ambulation	1,447 (37%)
Ambulation with crutch	245 (6%)
Ambulation with walker	1,702 (44%)
Take steps with support	258 (7%)
No ambulation	245 (6%)
Total	3,897 (100%)

**Table 3:** Subtypes of Cerebral Palsy (CP)

Subtypes of CP	Number of Patients
Spastic diplegia	3,076 (79%)
Spastic quadriplegia	513 (13%)
Spastic triplegia	247 (6%)
Spastic hemiplegia	61 (2%)
Total	3,897 (100%)

Spasticity damages the musculoskeletal systems in children and adults, and there is no evidence indicating the benefits of CP spasticity. Moreover, spasticity does not disappear spontaneously. Ideally, CP spasticity must be removed at an early stage of development. In our series of patients, SDR has removed CP spasticity permanently without adverse effects. Our preferred age for SDR is two to three years. We performed SDR on 1,089 children two to three years old and 1,660 on children four to six years old (Table 1). The advantages of SDR at a young age include ability to develop motor functions free of spasticity, decreased severity of functional decline during growth spurts, fewer deformities and orthopedic surgeries, and reduced burden of spasticity management.

We also performed SDR on 139 adults 18 to 49 years old. All of the adult patients sought SDR because of the symptoms of early aging (i.e., increasing body pain and a decline of strength, endurance, mobility, and balance). Nearly all patients selected for SDR were independent ambulators and received orthopedic surgery previously. In the adults, SDR reduced spasticity and resolved most symptoms of early aging (Videos 1, 2). They resumed better strength, endurance, balance, and quality of walking. Also, they experienced significantly reduced severe body pain and resumed outdoor activities including recreational sports. A few patients who received no postoperative rehabilitation failed to improve. The fact that the delayed SDR in adulthood can reverse the natural course of the early aging process is significant since adult population with spastic CP will increase in the future.

Concerning mobility before SDR, 1,447 of our patients walked independently without devices. We learned that children with mild spastic diplegia lose motor functions after seven to ten years of age. Those who walk independently in early childhood reported deteriorated motor function, which requires them to use a device to assist in walking as adults. SDR did not change the GMFCS Levels of the independent walkers, but the quality of walking, balance, sitting, and strength invariably improved. They started enjoying vigorous physical activities and participated in sports, which were impossible before SDR due to spasticity. Also, as shown in our recent studies, childhood SDR prevents early aging and eventual loss of independent walking as they approach 50 years of age.

For 30 years, we have been in contact with patients after SDR. Immediate postoperative complications are infrequent and treatable. Of our 3,897 patients, three patients developed a wound infection and received intravenous antibiotics treatment. Seven patients developed CSF leak and required surgical repair. In the long-term, SDR via single level laminectomy has been proven to be safe. Only two patients developed L1-T12 kyphosis and underwent spine fusion. A small portion of patients persisted with numbness and diminished sensation in small areas of the lower limbs. No patient developed urinary incontinence or muscle weakness directly related to rhizotomy. Overall, the major complications occurred in 0.3% of patients.

## Conclusions

There is sufficient evidence supporting the short and long-term benefits of SDR. The evidence indicates that SDR can reduce spasticity permanently and eliminate neurological complications in patients with spastic CP. Furthermore, while SDR through a multilevel laminectomy or laminotomy can cause spinal deformities, SDR through a single-level laminectomy can prevent spinal deformities. All the evidence points to overwhelming support for SDR as a treatment plan. We will continue to gather support for our claim that SDR can change the lives of patients forever.
